# Cerebello-cortical functional connectivity may regulate reactive balance control in older adults with mild cognitive impairment

**DOI:** 10.3389/fneur.2023.1041434

**Published:** 2023-04-17

**Authors:** Lakshmi Kannan, Tanvi Bhatt, Olusola Ajilore

**Affiliations:** ^1^Department of Physical Therapy, University of Illinois at Chicago, Chicago, IL, United States; ^2^Department of Psychiatry, University of Illinois at Chicago, Chicago, IL, United States

**Keywords:** fall risk, functional connectivity, cognitive decline, reactive balance responses, higher cortical cognitive function, cerebello-cortical networks

## Abstract

**Background:**

Older adults with mild cognitive impairment (OAwMCI) experience a two-fold increased risk of falling compared to their cognitively intact counterparts. This increased risk could be attributed to impairments in balance control mechanisms (both volitional and reactive), however, the exact neural substrates contributing to the balance impairments remain unclear. While changes in functional connectivity (FC) networks in volitional balance control tasks have been well highlighted, the relationship between these changes and reactive balance control has not been examined. Therefore, this study aims to explore the relationship between FC networks of the brain obtained during resting state fMRI (no visualization or active task performed) and behavioral measures on a reactive balance task in OAwMCI.

**Methods:**

Eleven OAwMCI (< 25/30 on MoCA, > 55 years) underwent fMRI and were exposed to slip-like perturbations on the Activestep treadmill. Postural stability, i.e., dynamic center of mass motion state (i.e., its position and velocity) was computed to determine reactive balance control performance. The relationship between reactive stability and FC networks was explored using the CONN software.

**Results:**

OAwMCI with greater FC in default mode network-cerebellum (r^2^ = 0.43, *p* < 0.05), and sensorimotor-cerebellum (r^2^ = 0.41, *p* < 0.05) network exhibited lower reactive stability. Further, people with lower FC in middle frontal gyrus-cerebellum (r^2^ = 0.37, *p* < 0.05), frontoparietal-cerebellum (r^2^ = 0.79, *p* < 0.05) and cerebellar network-brainstem (r^2^ = 0.49, *p* < 0.05) exhibited lower reactive stability.

**Conclusion:**

Older adults with mild cognitive impairment demonstrate significant associations between reactive balance control and cortico-subcortical regions involved in cognitive-motor control. Results indicate that the cerebellum and its communications with higher cortical centers could be potential substrates contributing to impaired reactive responses in OAwMCI.

## Introduction

Mild cognitive impairment (MCI) is a state of memory decline affecting about 3 to 42% of older adults above 65 years of age in the United States with at least 32% of them progressing to dementia within 5 years (1, 2). These older adults with mild cognitive impairment (OAwMCI) experience subtle balance control and gait deficits resulting in a two-fold increased risk of falls compared to their cognitively intact older adults (CIOA). Additionally, OAwMCI fallers are five times more likely to be admitted to the hospital compared to non-fallers. The consequences of such falls (like fractures, head injuries etc.) significantly affects their quality of life and independent functioning that increases financial and psychosocial burdens (3, 4). Considering the high incidence of falls among OAwMCI, a thorough comprehension of the underlying causative mechanisms of falls is imperative for designing effective fall prevention interventions.

There is enough evidence that OAwMCI exhibit significant alterations in structural (i.e., physical areas/tracts that connect brain/spinal cord regions) brain connectivity compared to CIOA (5–11). Such alterations are postulated to affect their physical function during activities of daily living like poor balance control and decreased walking function. Specifically, OAwMCI with lower gray matter volume in the temporal horn, frontal lobe, and caudate exhibited postural instability. People with medial temporal atrophy, reduced volume in hippocampus, middle frontal gyrus and superior frontal gyrus showed an increased fall risk. Furthermore, OAwMCI with increased number of white matter lesions in the frontal, parietal, and temporal cortices; thalamus, basal ganglia, and brainstem exhibited postural instability. People with reduced fractional anisotropy in corpus callosum, forceps minor, and left inferior fronto-occipital fasciculus had decreased gait speed when performed with a serial subtraction task. In the same study, reduced fractional anisotropy in the above-mentioned areas were also associated with increased number of falls (5–11).

Additionally, evidence shows disrupted functional connectivity (i.e., strength to which an activity between 2 or more cortical/subcortical signals correlates over time, FC) between networks that regulate motor and attentional demands in OAwMCI (12–14). Specifically, alterations in FC, within default mode network, frontoparietal network, supplementary motor area, dorsal attention network, sensorimotor network, executive control network, and salience network, have been established among OAwMCI (12–14). Studies demonstrate increased FC within default mode network during resting state in OAwMCI compared to healthy counterparts. The default mode network consists of medial prefrontal cortex, posterior cingulate cortex/precuneus, and inferior parietal lobe and the inability to deactivate this network during task performance is often associated with impaired cognitive function that could interfere with performance of goal-directed activities (15). A recent study showed that an increased default mode network activity at rest was associated with decreased dual task performance (simultaneous performance of motor and cognitive task) (12). Additionally, increased default mode network – supplementary motor area FC at rest was associated with decreased gait speed and increased postural sway (12). Similarly, OAwMCI fallers showed significantly lower default mode-sensorimotor network FC which was also associated with increased postural sway. Apart from this, studies show decreased FC within frontoparietal regions associated with decreased executive function (including motor planning and execution) and information processing (13, 14). While tasks in above-mentioned studies involve volitional activities, no study has examined the relationship of these networks with reactive balance control, activated to maintain or recover balance upon unexpected perturbations to the body.

Reactive balance responses are motor responses triggered upon an external perturbation to reestablish the relationship between the center of mass (COM) and base of support (BOS). These responses involve eliciting different strategies and further adjust the response based on the perturbation magnitude. Small magnitude perturbations elicit in-place strategies (using ankle or hip) in an attempt to restore the COM position within base of support. Large magnitude perturbations involve change in support strategies (i.e., stepping or reaching out) to reestablish larger BOS such that the displaced COM falls within it. Studies mainly use support surface movable platforms or motorized treadmills to deliver external perturbations for inducing balance loss in a safe environment. Pathologies related to neurocognitive disorders, such as MCI, when combined with age-related changes can significantly accelerate reactive balance control deficits (16, 17). For example, one study reported that performance on reactive balance control performance, measured using the BESTest, was significantly reduced among OAwMCI compared to CIOA (17). Our recent study demonstrated that upon exposure to stance forward-slip like perturbation, OAwMCI showed significantly decreased compensatory stepping responses compared to young adults and CIOA (16). This was demonstrated by OAwMCI having the lowest postural stability, shortest step length, slowest step initiation, and execution times (16). Further, they were unable to modulate (parametrize muscle and joint forces) their responses based on the perturbation magnitude (16). It should be noted that our study only examined and reported behavioral findings. Despite the high fall risk incidence among OAwMCI, studies have understudied the neural contributions to balance recovery that may be crucial for understanding the increased fall risk.

A neural control of compensatory stepping responses in reactive balance control is postulated to be triggered from subcortical balance control centers *via* the brainstem neural loop with cortical involvement only in the later phase of balance recovery *via* transcortical loop (18, 19). Higher cortical centers may play a critical role in the success of this response by contributing to modulate it based on prior experience and current task/environmental demands (20–22). Our recent findings indicate that structural brain integrity negatively influences reactive balance control such that OAwMCI with decreased fractional anisotropy in corticospinal, and frontopontine tracts showed reduced postural stability (23). Further, OAwMCI with reduced gray matter volume in brainstem and cerebellum exhibited reduced postural stability (23). Given such potential impact of the pathology associated structural brain changes in OAwMCI of reactive balance control, understanding how such changes affect the FC within/between cortico-subcortical regions may provide a better mechanistic understanding on the increased risk of falls in OAwMCI. Therefore, no hypotheses were formulated, and this study was entirely exploratory that examined the relationship between FC networks of the brain (i.e., between/within cortical and subcortical areas commonly associated with cognitive-motor functions) obtained during resting state fMRI while lying down in a scanner (no visualization or active task performed), and behavioral measures on a reactive balance control task in OAwMCI.

## Methods

### Participants

The study included eleven OAwMCI above the age of 55 years after obtaining a written informed consent. This study was approved by the University of Illinois at Chicago (UIC) institutional review board (Protocol #2018-1257). Participants were recruited by posting flyers at geriatric centers, nearby independent living senior centers, bus stops, train stations, and grocery stores.

### Participants' eligibility

To be included, participants must receive a score between 18 and 24/30 on the Montreal Assessment on Cognitive Assessment (MoCA). Participants with uncontrolled cardiovascular disease, presence of any neurological condition (e.g., Parkinson's disease, Alzheimer's disease), and/or severe musculoskeletal diseases that may interfere with the ability to undergo balance control testing were excluded. People with the inability to walk independently for more than 10 meters (to verify ambulatory status among community dwelling older adults) and with heel bone density T-score of < −2.0 (measured using Lunar Achilles Insight) indicating risk of osteoporosis were also excluded for safety of participants during the reactive balance test.

### Magnetic resonance imaging data acquisition

All imaging was acquired at the UIC Center for Magnetic Resonance Research using a 3 Tesla GE Discovery MR750 System (Milwaukee, WI) with a 32-channel head coil.

### Anatomic MRI

High resolution 1 mm isotropic voxel resolution T1-weighted (T1w) images were obtained using a 3D axial FSPGR BRAVO imaging sequence with the following parameters: slice thickness = 1 mm, in-plane resolution = 1 mm, repetition time (TR) = 9.3 ms, echo time (TE) = 3.8 ms, inversion time (TI) = 450 ms, flip angle = 13°, field of view (FOV) = 220 × 220 mm. This was used as structural volumes for preprocessing.

#### Resting state functional MRI

T2 weighted images were obtained for determining functional whole-brain blood-oxygen-level dependent (BOLD) and optimized to reduced susceptibility artifacts with TR = 2000 ms, TE = 25 ms, flip angle = 82°, FOV = 220 × 220 mm, acquisition matrix 64 × 64, slice thickness = 3 mm, gap = 0 mm, 44 axial slices, 182 volumes per run. High resolution T1w structural scan was performed for anatomical localization. Participants were made to lie in the scanner and were asked to focus on an “X” with open eyes on the screen for 8 min without thinking anything. Resting state fMRI data preprocessing and analysis was conducted using CONN toolbox (24) which uses statistical parametric mapping software (SPM 12). Realignment was performed to correct for motion, corrected for errors in slice timing, outlier detection was subjected, and co-registered to the anatomical image. These images were spatially transformed to standard MNI space using transformation, resampled to 2-mm voxels, and smoothed with an 8 mm FWHM Gaussian kernel prior to analysis as detailed in Nieto-Castanon (2020).

#### Reactive balance control task

The Active step (Simbex, Lebanon, NH) motorized treadmill was used to induce a stance perturbation. The full-body kinematics were recorded *via* Cortex software using an eight-camera motion capture system (Motion Analysis, Corporation, Santa Rosa, CA) with a sampling rate of 120 Hz. A safety harness attached to an overhead metal arch prevented participants' knees to contact the belt surface in case of a fall. Twenty-nine Helen Hayes markers were placed bilaterally on bony landmarks to compute the center of mass (COM), and one marker was placed on the treadmill belt to identify the perturbation onset (i.e., sudden forward treadmill belt acceleration). Participants attained a comfortable stance position with their feet shoulder-width apart. They were instructed to execute a natural response to regain their balance by taking a step upon a sudden forward movement of the belt (slip-like perturbation, prevalent type of accidental falls). A familiarization trial was provided before the actual test and participants were unknown to perturbation onset (acceleration = 21.5 m/s^2^, speed = 0.86 m/s, distance = 0.02m, duration = 40 ms). The reactive stability was computed using a custom-written algorithm in MATLAB version 2014b (The MathWorks Inc., Nactick, MA).

*Reactive stability* is calculated as the shortest distance of the relative COM state (i.e., its position and velocity) to the dynamic feasible theoretical boundary for backward loss of balance (25). The relative COM position was derived by expressing the absolute COM position relative to the rear edge of the base of support (BOS) normalized to each individual's foot length. Similarly, the COM velocity was expressed relative to the velocity of the rear BOS normalized by the factor √*g* × *bh*, where *g* is the gravitational acceleration and *bh* is the individual's body height (Note × indicates multiplication) (26). Higher values indicate greater stability.

### Statistical analyses

#### Regions of interest

**Figure 1:**
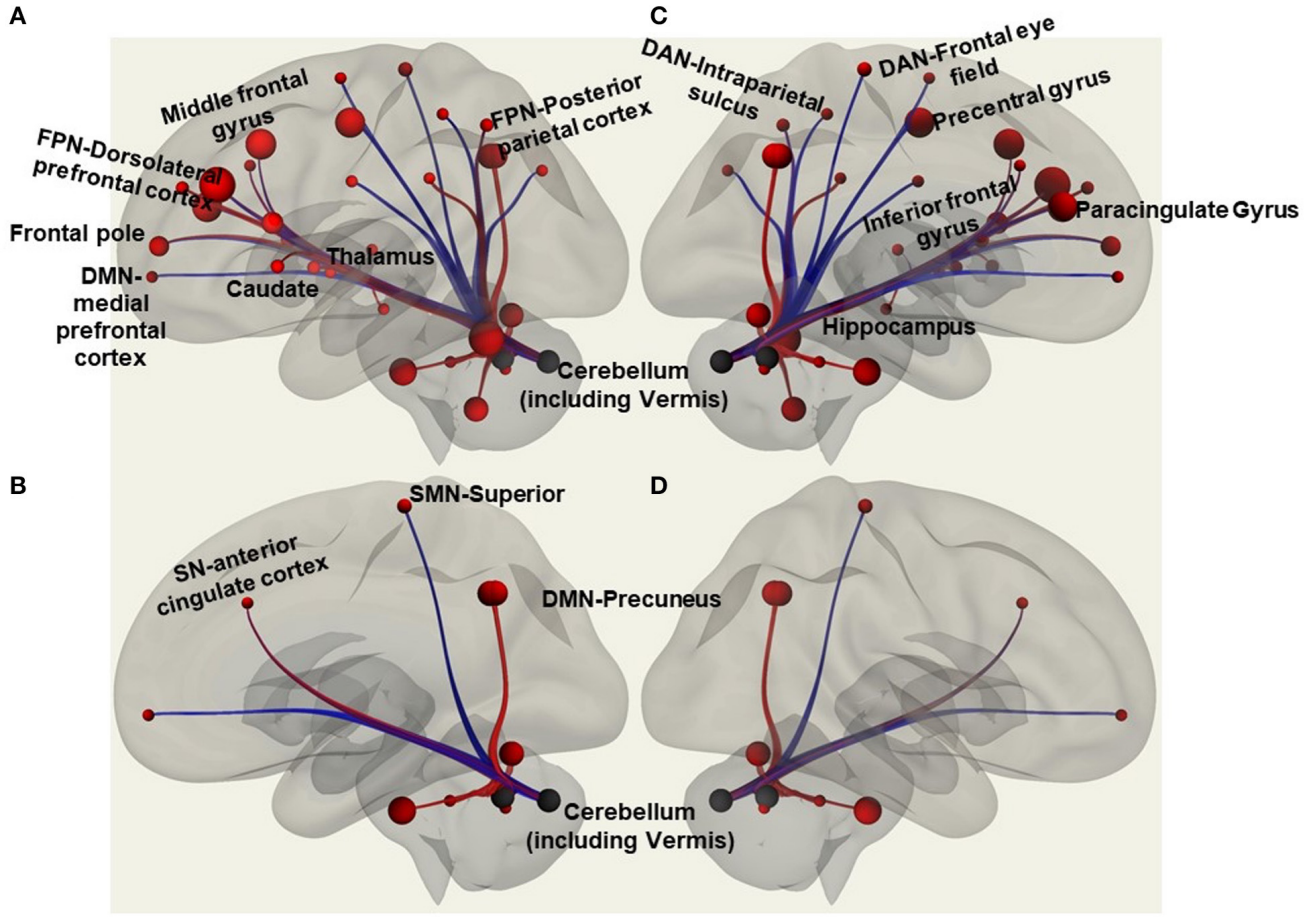
Regions of interest in the CONN software included functional connectivity analyses of atlas-atlas, altas-network, and network-network associations with red lines to indicate increased functional connectivity strength and blue lines to show decreased functional connectivity strength. The figure is a representation of some of those regions with **(A)** showing atlas regions involving frontal pole, middle frontal gyrus, caudate, thalamus, cerebellum; and network regions like frontoparietal network (FPN) that includes dorsolateral prefrontal cortex and posterior parietal cortex. **(B)** Shows atlas region involving the cerebellum; and anterior cingulate cortex of salience network (SN), superior of sensorimotor network (SMN), and precuneus of default mode network (DMN). **(C)** Shows inferior frontal gyrus, precentral gyrus, paracingulate gyrus, cerebellum and vermis, hippocampus; and frontal eye field, and intraparietal sulcus of dorsal attention network (DAN). **(D)** Shows precuneus of DMN and the cerebellum and vermis.

Functional connectivity (FC) is the statistical relationships between cortical/subcortical signals over time that defines the strength to which activity between cortical/subcortical correlates. Atlas-Atlas (FSL Harvard-Oxford atlas cortical and subcortical areas), Network-Network (Yeo Parcellation) and Atlas-Network resting state FC were explored for brain regions associated with balance control (Figure 1) for the resting state fMRI data collected. The atlas areas included Frontal pole (cognitive function), middle frontal gyrus, Precentral gyrus (balance control), Inferior frontal gyrus (motor inhibition, attention and working memory cognitive function), Paracingulate gyrus (motor function), Precuneus (balance control), Caudate (cognitive function), Putamen (motor learning and control), Pallidum (cognitive and motor processing), hippocampus (memory), Cerebellum (balance control), Vermis (balance control), and Brainstem (balance control). The networks included default mode (medial prefrontal cortex, posterior cingulate cortex/precuneus, and inferior parietal lobe), sensorimotor (primary motor cortex, cingulate cortex, premotor cortex, and supplementary motor area), salience (anterior cingulate cortex, anterior insula, right rostral prefrontal cortex), dorsal attention (visual motion area, frontal eye fields, superior parietal lobule, intraparietal sulcus, ventral premotor cortex), frontoparietal (dorsolateral prefrontal cortex, posterior parietal cortex), and cerebellar.

#### Resting state functional connectivity associations with behavioral measures

Reactive stability was inputted as a covariate into the CONN software to run a regression analysis for examining the resting state FC within and between regions of interest. A priori significance threshold of *p False discovery rate* = *0.2* was set to correct for multiple comparisons and these values are reported in the results.

We performed planned bivariate Pearson's correlation of all resting state FC networks (default mode, sensorimotor, salience, dorsal attention, frontoparietal, and cerebellar) with cerebellum, vermis, and brainstem to determine their relationship with reactive stability. For this, we used individual z-scores from CONN software exported after first level analyses. Further, to validate the robustness of our results, we performed a multiple comparisons test – False discovery rate (by comparing all the *p*-values) using the Benjamini and Hochberg (27) procedure.

## Results

### Demographics

A total of 11 participants included in the study analysis had a MoCA score ranging from 19 to 25 and their age, gender, height, and weight are provided in Table 1.

**Table 1:** Demographics and clinical characteristics of older adults with mild cognitive impairment (MCI).

	**OAwMCI**
Age [Means (SD)]	62.2 (5.9)
Range in years	56-74
Sex (M/F)	8/3
Arm Dominance (R/L)	11/0
Height (cm) [Means (SD)]	173.73 (8.95)
Range in cm	152.4–181.61
Weight (lbs) [Means (SD)]	162.62 (30.56)
Range in lbs	116–211
BBS Out of 56 [Means (SD)] Pre	54.18 (2.08)
Range	49–56
MoCA Out of 30 [Means (SD)]	21.63 (1.8)
Range	19–23
Reactive stability [Means (SD)]	−0.11 (0.39)
Range	−0.84–0.266

BBS, Berg Balance Scale; MoCA, Montreal Cognitive Assessment.

### Effect of stability on resting state Atlas-Atlas functional connectivity

**Figure 2:**
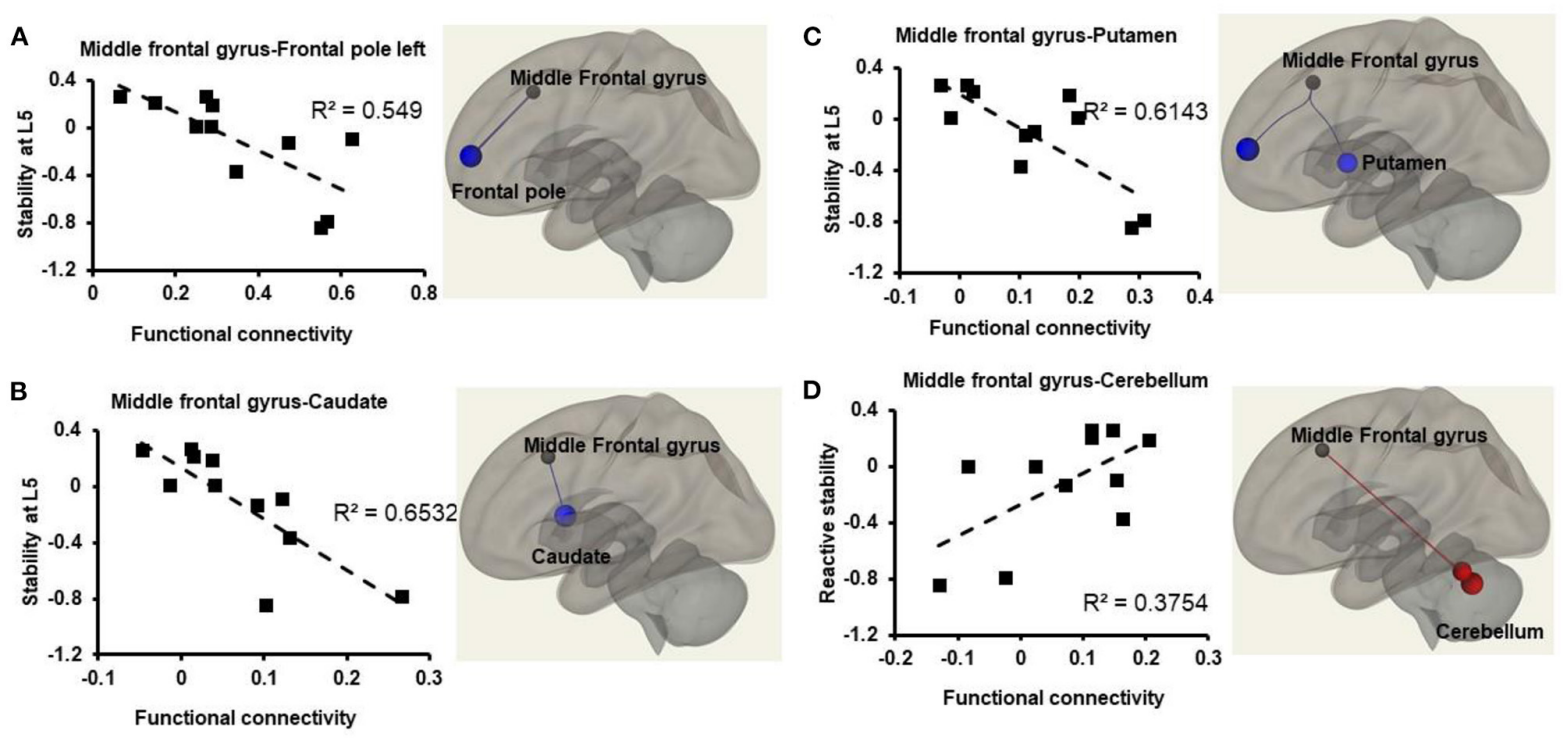
Relationship between atlas-atlas network (represented in z-scores on the x-axis) and reactive stability (represented on the y-axis) in older adults with mild cognitive impairment. Weakened connectivity in the brain is indicated via blue lines and increased functional connectivity with red lines. **(A)** Weakened functional connectivity between middle frontal gyrus and frontal pole left in the brain such that older adults with mild cognitive impairment with higher reactive stability exhibited lower functional connectivity strength during resting state. **(B)** Weakened functional connectivity between middle frontal gyrus and caudate in the brain such that older adults with mild cognitive impairment with higher reactive stability exhibited lower functional connectivity strength during resting state. **(C)** Weakened functional connectivity between middle frontal gyrus and putamen in the brain such that older adults with mild cognitive impairment with higher reactive stability exhibited lower functional connectivity strength during resting state. **(D)** Increased functional connectivity between middle frontal gyrus and cerebellum such that older adults with mild cognitive impairment with higher reactive stability exhibited higher functional connectivity strength during resting state.

**Figure 3:**
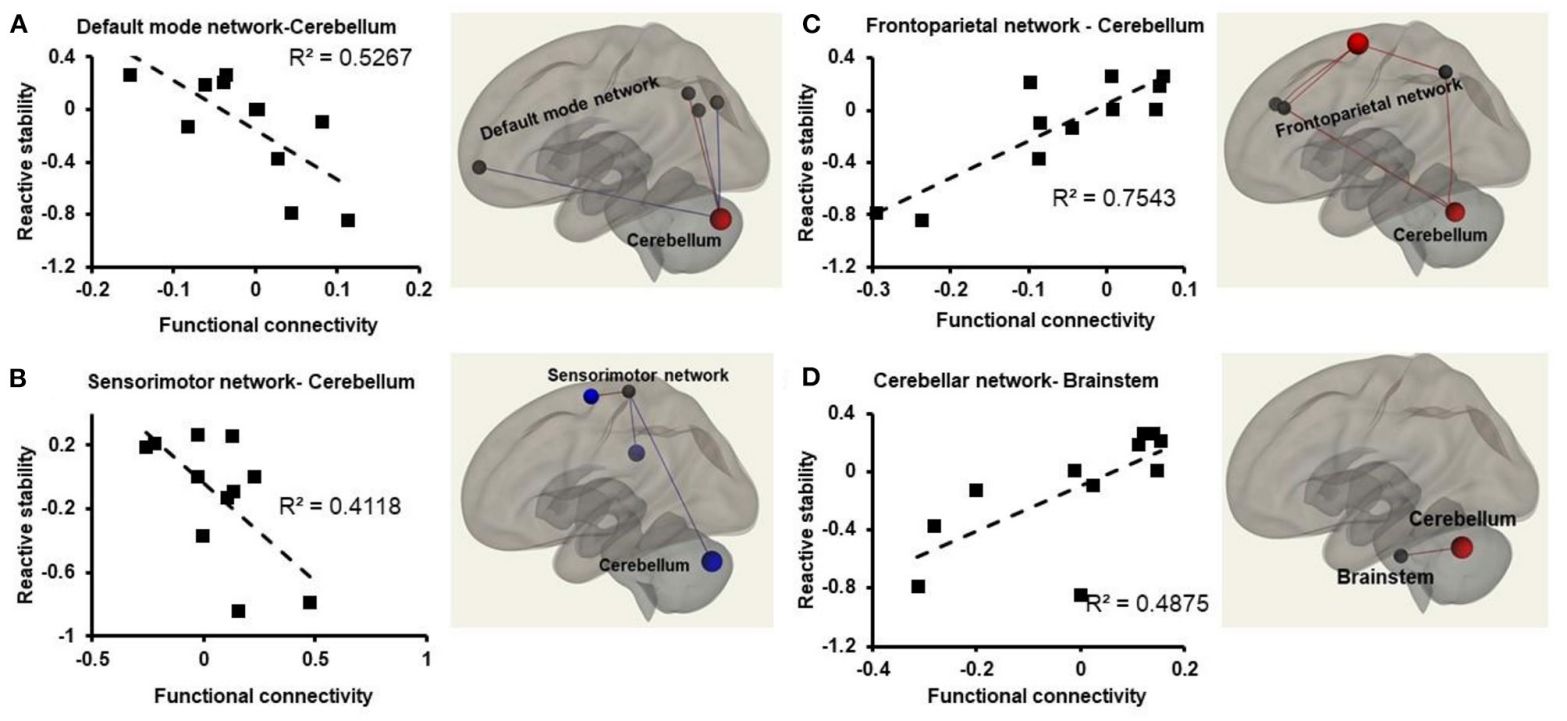
Relationships between atlas-network (represented in z-scores on the x-axis) and reactive stability (represented on the y-axis) in older adults with mild cognitive impairment. Weakened connectivity in the brain is indicated via blue lines and increased functional connectivity with red lines. **(A)** Weakened functional connectivity between default mode network and the cerebellum in the brain such that older adults with mild cognitive impairment with higher reactive stability exhibited lower functional connectivity strength during resting state; **(B)** weakened functional connectivity observed between the sensorimotor network and the cerebellum in the brain such that older adults with mild cognitive impairment with higher reactive stability exhibited lower functional connectivity strength during resting state; **(C)** increased functional connectivity strength observed between the frontoparietal network and the cerebellum in the brain such that older adults with mild cognitive impairment with higher reactive stability exhibited higher functional connectivity strength during resting state; **(D)** increased functional connectivity strength observed between the cerebellar network and the brainstem in the brain such that older adults with mild cognitive impairment with higher reactive stability exhibited higher functional connectivity strength during resting state.

We observed a significant decrease in FC at resting state between frontal pole left and middle frontal gyrus (β = −0.46, *p* = 0.02), middle frontal gyrus right and caudate (β = −0.23, *p* = 0.04), middle frontal gyrus and putamen (β = −0.24, *p* = 0.03) (Figures 2, 3). Additionally, we observed a significant increase in FC between middle frontal gyrus and cerebellum (β = 0.33, *p* = 0.04), middle frontal gyrus and Vermis (β = 0.38, *p* = 0.09), cerebellum and vermis (β = 0.48, *p* = 0.05) (Figure 2).

### Effect of stability on resting state network-network functional connectivity

We observed a significant decrease in FC between dorsal attention Frontal eye field left and dorsal attention intraparietal sulcus (β = −0.25, *p* = 0.02). Additionally, we observed a significant increase in FC between salience network and sensorimotor lateral network right (β = 0.38, *p* = 0.02) and left (β = 0.47, *p* = 0.02), dorsal attention Frontal eye field left and default mode lateral parietal (β = 0.28, *p* = 0.06).

### Effect of stability on resting state atlas-network functional connectivity

We observed a significant decrease in FC between cerebellum and sensorimotor network (β = −0.39, *p* = 0.2), vermis and sensorimotor network (β = −0.41, *p* = 0.006), vermis and default mode network medial prefrontal cortex (β = −0.29, *p* = 0.07). Additionally, we observed that there was a significant increase in FC between cerebellum and frontoparietal network (posterior parietal cortex) (β = 0.39, *p* = 0.04), cerebellum and frontoparietal network (lateral prefrontal cortex) (β = 0.37, *p* = 0.09), cerebellum and cerebellar anterior network (β = 0.44, *p* = 0.1), cerebellum and default mode network (posterior cingulate cortex) (β = 0.31, *p* = 0.07), accumbens and frontoparietal network (lateral prefrontal cortex) (β = 0.31, *p* = 0.13), vermis and cerebellar posterior network (β = 0.39, *p* = 0.18) (Figure 3).

### Planned associations between reactive stability and functional connectivity

#### Default mode network

A significant decrease in FC was observed between DMN-Cerebellum [r^2^ (10) =0.52, *p* = 0.01], and DMN-Vermis [r^2^ (10) =75, *p* = 0.001] such that people with higher reactive stability had lower FC between these regions (Figure 3A).

#### Sensorimotor network

A significant decrease in FC was observed between SMN and Cerebellum [r^2^ (10) = 0.41, *p* = 0.050], SMN and Vermis [r^2^ (10) = 0.57, *p* = 0.007] such that people with higher reactive stability had lower FC (Figure 3B).

#### Frontoparietal network

A significant increase in FC between FPN and cerebellum [r^2^ (10) = 0.75, *p* = 0.001] was observed such that people with higher reactive stability had higher FC (Figure 3C).

#### Cerebellar network

A significant increase in FC between CN-Cerebellum [r^2^ (10) = 0.78, *p* < 0.001], CN-Vermis [r^2^ (10) = 0.61, *p* = 0.004], CN-Brainstem [r^2^ (10) = 0.49, *p* = 0.01] (Figure 3D), was observed such that people with higher reactive stability had higher FC.

#### Multiple comparisons

False discovery rate for planned comparisons revealed that of the eight cases reported above, all of them were true for reactive balance control, thus validating the robustness of our findings.

## Discussion

This pilot based preliminary exploratory study was conducted to understand the association of reactive stability with FC between/within cortical and subcortical areas commonly associated with cognitive-motor functions among OAwMCI. Our study provides preliminary findings that altered (increase or decrease) FC of cognitive-motor related cortico-subcortical regions (observed during resting state fMRI) may contribute to the reduced reactive stability predisposing OAwMCI to increased fall risk. These results are specific to OAwMCI and future studies involving CIOA for a comparative analysis may yield better understanding of the involvement of the cortico-subcortical regions involved in reactive balance control.

### Atlas-atlas

Our results showed that the middle frontal gyrus located in the frontal lobe of the brain had weakened FC with caudate and putamen at rest such that OAwMCI with lower FC in these regions was associated with higher reactive stability. Based on MRI studies in young adults, activation of the middle frontal gyrus during tasks such as imagining or observing a slipping state, or observing a perturbed stance on a wobble, it is postulated that the middle frontal gyrus could be involved in planning and initiating movements when attention is reallocated to an unexpected stimulus (28–31). Caudate and putamen are parts of the basal ganglia, a key controller of motor sequence and modification of motor strategies and involved with higher-order cognitive aspects of motor control primarily regulating motor planning and execution (32–35). Specifically, during an external perturbation, it acts as a mediator between the motor cortex and brainstem *via* the basal ganglia-cortical loop (1 of 2 main central loops for postural response) for selecting a brainstem response during the initial state of balance loss (32–35). During an unpredicted balance loss, resources from the middle frontal gyrus and the basal ganglia could be recruited to relay and optimize pre-selected/triggered motor responses, i.e., compensatory stepping response. Therefore, it is possible that OAwMCI with higher FC in our study could not deactivate these areas at rest subsequently hindering the ability to trigger an appropriate response when the condition demands resources. Further, the ability to reallocate attentional resources (required for motor planning, step initiation, and execution to recover from balance loss), could have been inhibited among OAwMCI. It should be noted that this postulation is based on results yielded during a resting state condition that are subjected to change when a task-based fMRI is introduced. While task specific fMRI may be difficult to incorporate for reactive balance control, a mental imagery-based fMRI may provide a comprehensive understanding.

The middle frontal gyrus showed higher FC with the cerebellum, and the vermis (area that connects the 2 cerebellar hemispheres) such that people with higher FC exhibited higher reactive stability. It is postulated that the cerebellum could be involved in identifying movement error, online planning a stepping response based on the environment, and modulating motor responses to match the intended movement (28, 36–38). Such involvement relates to cerebellum interaction with the middle frontal gyrus *via* the cerebellar-cortical loop (2 of 2 main central loops for postural response) for processing sensory information to modulate motor programs based on prior experience. Another study has shown a reduced FC between the middle frontal gyrus and cerebellum during resting state in people with Alzheimer's disease compared to CIOA (39). It is possible that OAwMCI with higher FC were able to better process the sensory information relayed to the cerebellum and convey to middle frontal gyrus (supplementary motor area) for modulating the motor reactive response.

### Network-network

The frontal eye field and intraparietal sulcus nodes of the dorsal attention network showed lower FC such that people with lower FC within this network exhibited higher reactive stability. Both the nodes are primarily recruited when there is a need for voluntary shift of spatial attention and flexibility to allocate attentional resources for effective cognitive processing (40–42). In our study, OAwMCI who could deactivate this network during resting state exhibited higher reactive stability. Contrastingly, a strong FC between the frontal eye field and the default mode network was observed such that OAwMCI with higher FC exhibited higher reactive stability. It is known that default mode network activation uses attentional resources required for one's introspection at rest or when not actively engaged in a task (43, 44). When intending to perform a task, the default mode network deactivates, freeing up resources for the task at hand. Previous studies have shown efficient working memory were associated with strong anticorrelation between dorsal attention network and default mode network (i.e., increased dorsal attention network and decreased default mode network FC) during resting state (45–47). However, OAwMCI and people with Alzheimer's disease showed the opposite (48) where there was a weak anticorrelation strength between these regions, i.e., decreased dorsal attention network and increased default mode network FC, which is in line with our results. Another study reported that executive function may be involved in motor planning for selecting the stepping strategy in response to an external perturbation (49). Therefore, the associations between dorsal attention network and default mode network observed in our study could suggest that cognitive state may influence reactive responses.

The results also showed a higher FC between the salience and sensorimotor networks such that OAwMCI with higher FC showed higher reactive stability. Regions of the salience network include anterior cingulate cortex, anterior insula and prefrontal cortex that is postulated to play a significant role in reactive balance control. Specifically, an earlier study showed that the anterior cingulate cortex and prefrontal cortex were activated during imagined slipping (28) and are thought to be involved in monitoring error during task performance and identifying balance loss. Additionally, the prefrontal cortex might help modulate balance recovery response based on the threat perceived in relation to the current body state (by anterior insula). On the other hand, the sensorimotor network comprises of primary motor cortex, cingulate cortex, premotor cortex, and supplementary motor area that are involved in modifying the ongoing movement. However, the relationship between salience network and sensorimotor network among OAwMCI compared to CIOA are not well known.

### Atlas-network

#### Cerebellum and default mode network

In line with previous findings, our study demonstrated that OAwMCI had reduced FC between the cerebellum and default mode network at rest (50). Additionally, it was observed that OAwMCI with lower FC between the cerebellum (including the Vermis) and default mode network exhibited higher reactive stability. This is the first study to explore the relationship of reactive balance control with default mode network and cerebellum. The cerebellum consists of three functional areas namely the spinocerebellum (proprioceptive information), the cerebrocerebellum (movement planning), and the vestibulocerebellum (orientation of head in relation to the body). The spinocerebellar tract provides online proprioceptive information about body segments to the cerebrocerebellum. It is postulated that these areas are involved in generating an “error signal” between the expected and the actual postural status, which is then conveyed to the task-specific internal models (gait vs. stability regulation) to formulate and initiate the motor action – such as triggering the compensatory stepping response (20–22, 51). While these functions relate more toward spinocerebellum and cerebrocerebellum, the specific involvement of vestibulocerebellum in reactive responses is not much highlighted. Recent findings indicate that proprioception and vestibular systems are impaired in OAwMCI, however, the severity is less compared to people with Alzheimer's disease (52). Additionally, people with vestibular loss had 3 to 4 times increased likelihood of having cognitive impairment compared to healthy controls (53). However, participants involved in our study were not assessed for vestibular function to understand its contribution in reactive responses.

#### Cerebellum and sensorimotor network

The precentral gyrus (motor) and postcentral gyrus (proprioception) and supplementary motor area (complex motor planning) of the sensorimotor network showed lower FC with the cerebellum (including vermis). Regardless of the older adults' cognitive state, a previous study observed that fallers demonstrate a higher FC within the sensorimotor network (54). Such increased FC was attributed to the scaffolding theory of aging and cognition (55), where an adaptive compensatory mechanism is enabled to counteract the age-associated disruption in the sensorimotor networks so that existing motor and/or cognitive processes can still be functional (54). When such adaptive compensatory mechanisms are operating at their full capacity for maintaining all daily living functioning, they cannot be recruited for flight or fright responses that are encountered suddenly such as a perturbation, resulting in a balance loss or fall. In line with this, our previous study showed that OAwMCI exhibited significantly deteriorated ability to initiate a compensatory stepping response when an external perturbation was induced (16). Therefore, it could be that OAwMCI who suffer significant brain atrophy may not have the ability to counteract the disrupted networks to generate cortico-subcortical networks required for effective reactive responses. This could have resulted in lower FC in the cerebellum and sensorimotor network (54).

#### Cerebellum and frontoparietal network

Our study observed a higher FC between the cerebellum and frontoparietal network, that includes dorsolateral prefrontal cortex (regulates cognitive function like executive function) and posterior parietal cortex (spatial attention). Additionally, OAwMCI with higher FC between these regions exhibited higher reactive stability. It has been postulated that the cerebellum could be involved in identification of balance loss and recovery (20). In line with this, our recent findings showed that reduced gray matter volume to be associated with reduced reactive balance control against backward loss of balance in OAwMCI (23). It could be that the termination of motor pathways resides in parts of cerebellum making it a critical area responsible for relaying balance recovery information (23). Additionally, studies have shown its role in regulating executive function based on anatomical links with sensorimotor, prefrontal, and parietal cortices that serve higher cognitive functions (56–58). Specifically, the dorsolateral prefrontal cortex along with the cerebellum is shown to be coactivated under non-motor working memory tasks (59). Such coactivation increases as the load of the cognitive task increases, thus increasing information processing. Additionally, because of anatomical cerebellar-cortical links, cognitive function is facilitated by learning from previous experiences (*via* the cerebellum role in adaptation) to accurately predict the occurrence of an upcoming threat and updates the sensitivity to perceive the threat for better prediction in the future. Thus, in the occurrence of a balance threat, which could be like the type of threat already stored in the memory by the dorsolateral prefrontal cortex (28, 60), would be required to retrieve the previously learned motor memory for triggering a response. However, the activation of dorsolateral prefrontal cortex is reduced during memory retrieval in OAwMCI (61). In line with this, our study showed that people with lower FC within the cerebellum, and between the cerebellum and frontoparietal network exhibited lower reactive stability.

**Figure 4:**
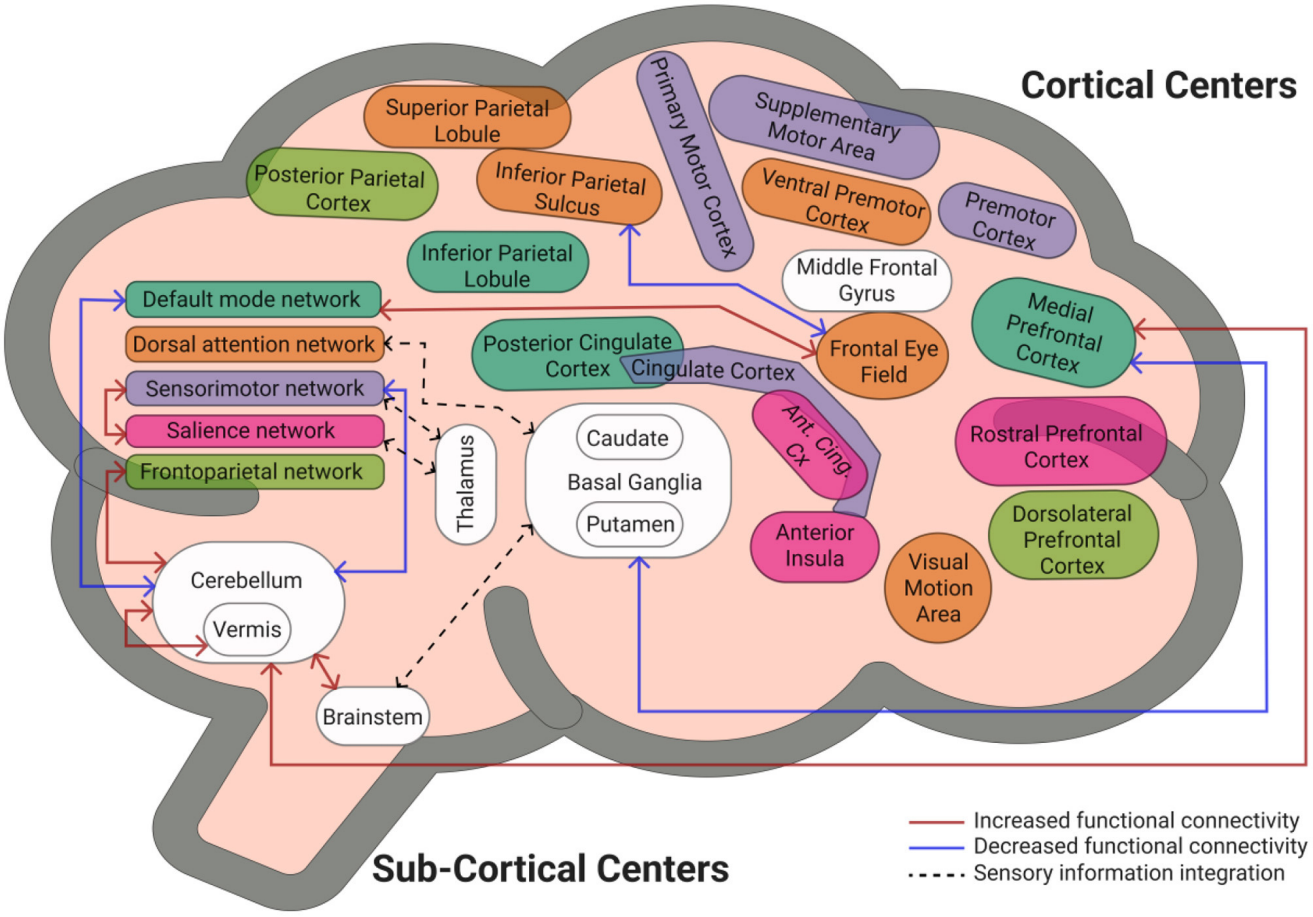
Summary model representing functional connectivity patterns across brain regions and that observed within/between the cerebellum and higher cortical networks/regions. The sensorimotor and salience network along with the brainstem communicate with the thalamus and basal ganglia to integrate sensory information perceived (denoted by black dotted lines). Our results indicate that the cerebellum could be the common center for relaying this sensory information. However, at a state of cognitive pathology like mild cognitive impairment causing uninterrupted functional activation/deactivation (denoted by red, increased functional connectivity strength; blue line, decreased/weakened functional connectivity), there is limited resources available for generating an “error signal” to even identify/perceive a balance loss and delay information processing.

Overall, our results support the existing evidence on FC patterns within/between brain regions in OAwMCI with preliminary evidence that these FC pattern may negatively influence balance recovery responses against unexpected external perturbations. It can be postulated that the cerebellum could be the common center for relaying perturbation specific information (acceleration, displacement, and velocity) to the higher cortical centers (regions involved in frontoparietal, sensorimotor, and default mode networks). Therefore, it could be that people with relatively preserved FC patterns may deactivate or activate corresponding networks online subsequently allocating and recruiting attentional resources to trigger an appropriate and effective response (Figure 4). However, it can be speculated that when uninterrupted functional activation/deactivation is ongoing due to a cognitive pathology, there is limited resources available for generating an “error signal” to even identify/perceive a balance loss and delay information processing. Such delay incapacitates the ability to simultaneously retrieve the motor memory (if any stored) further delaying triggering a compensatory response against an external perturbation while the COM at this time may already have moved farther from the BOS resulting the individual experiencing a fall. While this is just a postulation specific to OAwMCI, future studies should focus on mental imagery-based brain imaging along with a control group to yield a profound understanding of FC pattern associated with reactive responses.

The results demonstrating an association of the strength of resting state functional brain networks with reactive stability during stance perturbations could provide preliminary understanding of brain regions involved in balance recovery. However, the study findings must be interpreted in the light of its limitations. This study was a preliminary exploratory study and hence had a sample size of *N* = 11. Higher sample size may yield better conclusive findings. It should also be noted that OAwMCI included in our study were not assessed for their fall-risk and the inferences made from the results of this study might not be disease specific and could be markers of general characteristics of people with increased risk of falling. Secondly, the study included only OAwMCI, and inclusion of a CIOA may help interpret the increased/decreased FC in specific regions to provide better understanding of FC associated with reactive balance control. Future studies that include a control group could also use these FC patterns as preliminary source of networks to advance the understanding of reactive balance control. Additionally, the current study was conducted during resting state and analyzing FC pattern during a reactive balance control mental imagery task-based fMRI may yield a comprehensive understanding of the neural networks involved during reactive balance control. Thirdly, the included OAwMCI were not assessed for vestibular function to rule out its contribution that could affect reactive stability during an external perturbation. Lastly, given the probable conversion from mild cognitive impairment to Alzheimer's disease, future studies must consider type of MCI as a subgroup or covariate to determine if they affect the associated FC patterns.

## Conclusion

The results demonstrated statistically significant associations between measures of reactive measures and the cortico-subcortical regions contributing to cognitive and motor functions in OAwMCI. Specifically, higher reactive stability was associated with decreased FC in default mode and sensorimotor networks, and increased FC in cerebellar, and frontoparietal networks, thus, indicating higher fall risk. The study findings indicate that the cerebellum and its communications with higher cortical centers could be potential substrates contributing to impaired reactive responses in OAwMCI.

## Data availability statement

Data sharing requests will be furnished with a data-sharing agreement approved by the University of Illinois at Chicago institutional review board that contains commitments to 1) Using the data for research purposes only (no commercial use of the data), 2) not attempting to re-identify any participant, 3) securing the data using appropriate technology, and 4) destroying or returning the data after analyses. Other stipulations may be added to the data-sharing agreement if deemed necessary. Data may be shared as a complete or partial dataset depending on the request. Requests to access the datasets should be directed to the corresponding author.

## Ethics statement

The studies involving human participants were reviewed and approved by the Institutional Review Board at University of Illinois at Chicago. The patients/participants provided their written informed consent to participate in this study.

## Author contributions

LK: conceptualization, formal analysis, investigation, writing—original draft, writing–review and editing, visualization, and project administration. TB: conceptualization, writing—original draft, writing—review and editing, supervision, resources, and funding acquisition. OA: formal analysis and writing—review and editing. All authors contributed to the article and approved the submitted version.
